# Personality Traits as Predictors of Academic and Work Engagement in a Sample of Nursing Students and Professionals

**DOI:** 10.3390/nursrep15050174

**Published:** 2025-05-15

**Authors:** Maja Kućar, Andreja Brajša-Žganec, Marijana Neuberg

**Affiliations:** 1 Institute of Social Sciences Ivo Pilar, 10000 Zagreb, Croatia; 2 Department of Nursing, University Centre Varaždin, University North, Ul. 104. brigade 3, 42000 Varaždin, Croatia

**Keywords:** personality, nursing, work engagement, academic engagement, medical professionals

## Abstract

**Background/Objectives**: Academic engagement (AE) and work engagement (WE) are important indicators of performance and well-being in educational and occupational settings. Although these constructs are well researched independently, few studies have examined them concurrently among individuals navigating both academic and professional demands. Nursing students who are simultaneously employed as nurses represent a unique and under-researched population in this context. Understanding how personality traits influence both AE and WE in this dual-role group may offer insights relevant for academic success and well-being in healthcare settings. **Methods**: The sample consisted of 230 nursing students from a public university in Croatia who were also working as nurses. This study employed a repeated cross-sectional descriptive predictive research design (2022–2025). The participants completed questionnaires (UWES-9, UWES-S-9, and IPIP-15) during their university lectures. **Results**: The analysis yielded two personality clusters (adaptive—lower levels of neuroticism and higher levels of the other personality traits and maladaptive—the opposite). The participants in the adaptive cluster had higher levels of WE and AE. Regression analysis revealed that conscientiousness was a significant predictor for WE and AE, whereas agreeableness was a significant predictor for only WE. **Conclusions**: The findings highlight the importance of personality traits when addressing AE and WE, and fostering traits such as conscientiousness and agreeableness may enhance positive work and academic outcomes. Personality traits showed similar patterns of association with both AE and WE, indicating that dispositional factors may play a more crucial role in WE and AE than external influences.

## 1. Introduction

According to Schaufeli [1], work engagement (WE) involves a sustained, positive cognitive and emotional state, encompassing vigor, dedication, and absorption. Vigor entails high levels of energy and mental resilience during work, along with a readiness to invest effort and persevere through work challenges. Dedication involves a profound sense of involvement in one’s work, coupled with feelings of significance, enthusiasm, inspiration, and a readiness to tackle challenges. Lastly, absorption describes a state of deep concentration and contentment in one’s work. WE has commonly been conceptualized as a contrasting state to burn out in the realm of occupational psychology. It refers to a state where individuals exhibit high levels of energy, involvement, and efficacy in their work, whereas burnout is characterized by exhaustion, cynicism, and reduced professional efficacy. Many researchers have shifted their focus from burnout to the exploration of factors contributing to the desired outcome in the professional context—WE [1,2,3].

The theoretical and empirical research on WE has been translated to the academic context, so the term academic engagement (AE) has been coined. One can experience similar states (vigor, dedication, and absorption) but in relation to studying, attending lectures, or completing coursework [4]. The term AE is sometimes used synonymously with study engagement [5]. However, it is worth noting that ‘study’ may apply to all levels of education and ‘academia’ is usually used in relation to tertiary, higher education. AE is manifested in various student behaviors, such as their perception of the worth of the educational journey, attending classes, studying beforehand, completing assignments, and engaging in school events [6]. A systematic review revealed that higher AE is related to a plethora of positive outcomes such as higher self-efficacy, positive relationships or general well-being [7].

There are many potential correlates of both WE and AE, one of which is personality traits. Personality is usually defined as the unique and typical pattern of thoughts, feelings, and behaviors that distinguishes one individual from another. It is widely accepted that five major personality domains describe the personality profile of an individual [8]. The Big Five personality theory, also known as the Five-Factor Model (FFM), posits that personality can be summarized using five broad dimensions: openness to experience, conscientiousness, extraversion, agreeableness, and neuroticism, often found under the acronym OCEAN [9]. Conscientiousness is a personality trait that is most often mentioned in the context of WE or AE, motivation, performance, and work/performance context in general. Empirical research typically finds that higher levels of conscientiousness predict various desired outcomes in the work and academic spheres [8]. O’Connor and Paunonen [10] conducted a meta-analysis and revealed that conscientiousness has the strongest and most consistent correlation with academic success, among all personality traits. Komarraju et al. [11] reported that conscientiousness negatively predicts intrinsic and extrinsic motivation and general amotivation among college students. It was also found that agreeableness was negatively associated with amotivation, openness was positively associated with intrinsic motivation, and extraversion was positively linked to extrinsic motivation.

Personality dimensions are typically considered independent and are viewed individually, but advancements in personality research have led to an increase in studies that examine personality profiles [12,13,14]. A personality profile consists of a unique constellation of traits (e.g., high neuroticism combined with low scores on all four other dimensions). This different methodological approach allows for a holistic view of an individual and a deeper understanding of how individuals, rather than traits, interact within their environments. The cluster analysis approach can be labeled as a person-centered approach to personality, which is different to the variable-centered approach. Variable-centered models focus on isolated traits and their unique relationships with outcomes, whereas person-centered approaches aim to identify homogeneous subtypes of individuals on the basis of their personality profile. A study by Fisher and Robie [15] revealed three latent profiles of the four basic personality traits and called them maladaptive, adaptive, and highly adaptive. Openness to experience was excluded because it did not differ between profiles. Compared to the other two profiles, the maladaptive personality profile had the highest neuroticism and lowest extraversion, agreeableness, and conscientiousness. The highly adaptive profile had the lowest neuroticism and highest extraversion, agreeableness, and conscientiousness, while the adaptive profile had medium values (between maladaptive and highly adaptive) of all four personality traits. The participants with more adaptive profiles had higher levels of job satisfaction, job self-efficacy, and passion towards work. A study focused on various indicators of nursing professionals’ work performance reported a positive relationship between conscientiousness, openness, extraversion, and work outcomes such as work proficiency, adaptivity, and proactivity at the individual, team, and organizational levels [16]. Similarly, another study reported that openness, conscientiousness, and extraversion were positively related to nursing professionals’ WE [17]. A study that aimed to determine predictors of nursing professionals’ WE [18] revealed that higher levels of extraversion, agreeableness, conscientiousness, openness and lower levels of neuroticism positively predict all three subscales of WE (vigor, absorption, and dedication). A similar pattern of results was obtained in a study that examined the relationships between nursing students’ personality traits and different subscales of AE [19]. Their results suggest that students with higher levels of extraversion, agreeableness, conscientiousness, openness and lower levels of neuroticism experience more AE and their facets, vigor, dedication, and absorption. Jurado et al. reported similar results in a large sample of Spanish nursing professionals [20]. Participants with lower levels of neuroticism and higher levels of the other four personality domains tended to experience more vigor, dedication, and absorption. In all three cases [18,19,20], cluster analysis revealed two personality clusters in a sample of nursing professionals—participants in one cluster had lower levels of neuroticism and higher levels of the four other dimensions, while participants in the other cluster had higher levels of neuroticism and lower levels of the other four personality dimensions. A detailed explanation for this repeating pattern of results has not been thoroughly discussed in published studies, but it may be partially explained with the maladaptive–adaptive personality explanation proposed by Fisher and Robie [15].

Similarly to WE, research consistently shows that certain personality traits are strong predictors of AE. Conscientiousness, openness to experience, and extraversion have been found to be positively related to AE in various studies [11,21,22]. These traits are associated with characteristics such as self-discipline, curiosity, and sociability, which are likely to enhance students’ involvement in their academic pursuits. However, the relationship between neuroticism and AE is less clear, with some studies finding a negative association [23] and others reporting no significant correlation [21,24,25]. Interestingly, Komarraju et al. [11] found a weak, positive relationship between neuroticism and academic motivation. Further research is needed to fully understand the impact of neuroticism on AE. Mahama et al. [25] reported that the traits openness, extraversion, conscientiousness, and agreeableness positively predicted all aspects of AE (behavioral, emotional, and cognitive), while neuroticism was not a significant predictor of any of the AE dimensions.

Although many studies have explored WE [18] or AE [19] separately and in various contexts, to our knowledge, no study has explored both WE and AE in one sample of nursing professionals. There is a lack of research exploring both forms of engagement simultaneously, especially in populations that have dual roles. Employed nursing students represent a unique group for whom both work and academic engagement are vital for success and well-being. The lack of such research is understandable considering that students who are simultaneously employed are rarer than those who are solely working or studying. The objective of this study was to identify personality profiles among employed nursing students and determine whether they differ in terms of AE or WE. Additionally, the aim is to explore the relationship between various personality traits (openness, agreeableness, neuroticism, extraversion, and conscientiousness) and engagement levels in both academic and professional contexts.

### Research Questions

RQ1: Can distinct personality-based profiles be identified among employed nursing students using cluster analysis based on the Big Five personality traits?
RQ2: Do the identified personality-based clusters differ significantly in terms of WE and AE?
RQ3: To what extent do personality traits uniquely predict WE and AE after controlling for age and gender?

## 2. Materials and Methods

### 2.1. Study Design

This study employed a repeated cross-sectional descriptive predictive research design. Data were collected from spring 2022 to early 2025.

### 2.2. Participants

All participants in this study were enrolled in the professional undergraduate study of nursing or university graduate study of nursing—management in nursing (University North, Croatia), but were also employed full-time as nurses. In Croatia, individuals can be employed as licensed nurses after completing secondary education for nursing (high school). In total, 230 nursing students completed the survey (M_age_ = 31.23, SD = 7.47), of which 38.3% were bachelor’s-level students and 61.7% were master’s-level students. The majority of the study participants were female (86.1%). Due to work obligations, 80.9% of our sample are self-funded students which means they have less obligations regarding class attendence compared to state-funded, full-time students. Given that the participants were selected from one university where the authors have teaching obligations, the sample is convenient. A post hoc power estimation suggests that this study had lower power to detect small, standardized effects in the regression analysis, indicating that nonsignificant findings should be interpreted with caution. In contrast, the MANOVA test was well powered to detect large effects, supporting the robustness of the results.

### 2.3. Instruments

The IPIP-15 scale was used to assess the participants’ personality traits [26,27]. The scale is a short version of the International Personality Item Pool (IPIP) of the Big Five Personality Questionnaire. It measures the Big Five personality traits—extraversion (E), agreeableness (A), conscientiousness (C), neuroticism (N), and openness (I). In this shortened version of the questionnaire, there are three items per personality trait with a corresponding 5-point, Likert-type scale ranging from 1 (very inaccurate) to 5 (very accurate). The results for each trait were calculated as an average of the three corresponding items. The factor analysis yielded a clear five-factor structure, corresponding to the original Big Five model [7]. In this study, we use the term ‘openness,’ but we acknowledge that the three items primarily assess the intellectual component of openness (e.g., knowledge, vocabulary, and engagement with intellectually challenging material). The internal consistency of the factors (Cronbach’s α) ranged from 0.56 to 0.65 (original research—from 0.59 to 0.78). The slightly lower reliability coefficients observed in this study, compared to the original research, may be explained by the larger sample size used in the original validation study (N = 1248). Since each trait consists of only three questions, and reliability analysis is sensitive to the number of items, lower values are deemed acceptable and correspond to the original research [27,28,29]. Factor analysis for IPIP-15 was carried out. An exploratory factor analysis yielded five distinct factors with eigenvalues greater than 1, which is in line with theoretical expectations. In total, the five factors explained 64.76% of the total variance. Six items had significant cross-loadings on another factor, but in all the cases, the highest loading was on the expected factor. For example, the item ’I make friends easily’ had the highest load on extraversion but also loaded on agreeableness and openness.

The Utrecht Work Engagement Scale (UWES-9) is a self-assessment scale composed of nine items aimed at evaluating WE [30]. The items are answered on a Likert-type scale ranging from 0 (never) to 6 (every day/all the time). The instrument provides a total score and partial, factor scores for three dimensions of the WE (vigor, dedication, and absorption). The total and partial scores were calculated as an index—the sum of the items was divided by the number of the items. A higher total score reflects higher WE, and a higher partial score reflects higher vigor, dedication, or absorption. The internal consistency of the UWES-9 scale total score (Cronbach α) was 0.93, while subscales ranged from 0.85 to 0.96, which is considered to show high reliability. Schaufeli et al. [31] reported the reliabilities of the subscales across 10 countries—vigor (median α = 0.77), absorption (median α = 0.78), and dedication (median α = 0.85)—and the reliability of the total score varied between 0.85 and 0.92. An exploratory factor analysis identified a clear, one-factor solution. One factor explained 68.17% of the variance, so further analysis used the UWES-9 total score, not the three dimensions.

The Utrecht Work Engagement Scale—student version (UWES-S-9) was used to evaluate participants’ AE [32,33]. The items are answered on a Likert-type scale ranging from 0 (never) to 6 (every day/all the time). The instrument provides a total score and partial, factor scores for three dimensions of AE (vigor, dedication, and absorption). The total and partial scores are calculated as an index, and the sum of the items is divided by the number of items. A higher total score reflects higher AE, and a higher partial score reflects higher vigor, dedication, or absorption. The internal consistency of the UWES-S-9 scale (Cronbach α) total score was 0.96, while the subscales ranged from 0.89 to 0.93, which is considered to show high reliability. The reliabilities are somewhat higher than in the original study on UWES-S-9 where they ranged from 0.70 to 0.76, while 0.84 was the value of Cronbach α for the total score [3,33]. An exploratory factor analysis identified a clear, one-factor solution. One factor explained 75.59% of the variance, so further analysis used UWES-9 total score, not the three dimensions.

### 2.4. Procedure

The questionnaire was in digital format (Google forms—online survey) but was administered to students onsite during their university lectures. The participants used their phones to answer the study questions. One of the researchers was present during the questionnaire administration, which lasted for 15–20 min and was completed on a voluntary basis. Other than the questionnaires used in this study, participants also assessed 11 questions about their sociodemographic data (age, gender, employment type, etc.). The participants did not receive compensation for their participation. This study was conducted in accordance with the Declaration of Helsinki and approved by the Institutional Review Board (Ethics Committee) of Social Sciences Ivo Pilar (protocol code 11-73/22-2381, 23 May 2022).

### 2.5. Data Analysis

Descriptive statistics were computed to summarize the main study variables, and Pearson correlations were used to examine bivariate relationships between the main study variables. Cluster analysis was conducted to identify personality-based profiles among participants. To compare differences in WE and AE between the identified clusters, a multivariate analysis of variance (MANOVA) was performed. Additionally, hierarchical regression analyses were conducted to examine the unique contributions of personality traits to WE and AE while controlling age and gender. The assumptions for MANOVA and regression (e.g., normality, the homogeneity of variance–covariance, and multicollinearity) were checked before conducting the analyses. Normality was inspected for all main study variables based on histograms, Q-Q plots, skewness and kurtosis. While most variables showed no significant deviations from normality, agreeableness exhibited a slight skew towards higher values. Specifically, it showed a negative skewness and a kurtosis, indicating a distribution that was tilted towards higher values. All statistical analyses were performed using SPSS software, version 25 design.

## 3. Results

In total, 230 nursing students completed the survey (Mage = 31.23, SD = 7.47), of which 38.3% were bachelor’s-level students and 61.7% were master’s-level students. Each participant completed the survey only once (2022—N = 102, 2023—N = 50, 2024—N = 47, 2025—N = 31). The variation in sample size each year reflects fluctuations in the number of enrolled students each academic year and their attendance on the day of data collection. The majority of the study participants were female (86.1%). The descriptive statistics for the main study variables are shown in Table 1.

The relationships among the study variables at the bivariate level are shown in Table 2. The correlation between the outcome variables is positive (r = 0.59, *p* < 0.01) and moderate [34]. Regarding the relationship between outcome variables and personality traits, WE has the highest correlation with agreeableness (r = 0.33, *p* < 0.01) but also showed a significant, positive relationship with extraversion, conscientiousness, and openness and a negative relationship with neuroticism. On the other hand, AE showed the highest correlation with conscientiousness (r = 0.27, *p* < 0.01). It also correlates positively with extraversion, agreeableness, and openness and negatively with neuroticism.

### 3.1. Cluster Analysis

To identify personality profiles and determine whether there are statistically significant differences among profiles in WE and AE among nursing students, cluster analysis was performed. Cluster analysis is the formal study of methods and algorithms for grouping objects or participants according to certain measured or perceived characteristics or similarities. The aim of clustering is to find the structure and patterns in data and is usually exploratory in nature [35]. In this case, cluster analysis was performed based on five personality variables. The analysis revealed a fair clustering quality based on the average silhouette measure of 0.40 [36,37]. The silhouette score quantifies how similar an object is to its own cluster (cohesion) compared to other clusters (separation). It ranges from −1 to 1, where a score closer to 1 indicates that the object is well matched to its own cluster and poorly matched to neighboring clusters, suggesting a good clustering result. The clustering method separated 136 participants in cluster 1 and 94 participants in cluster 2. According to Fisher and Robie’s terminology [15], cluster 1 can be described as adaptive and cluster 2 as maladaptive—participants with higher levels of extraversion, agreeableness, conscientiousness, openness and lower levels of neuroticism belong to cluster 1, while participants with higher neuroticism and lower levels of all other four traits belong to cluster 2. The means and standard deviations for each personality trait in the clusters are shown in Table 3 as are the levels of personality traits in the total sample.

The order in which the traits are presented in Table 3, from conscientiousness to neuroticism, reflects their significance as input variables in cluster analysis. In this context, conscientiousness emerged as the most influential trait in distinguishing between clusters. Notably, the largest absolute difference between the means of cluster 1 and cluster 2 is observed for conscientiousness, while the smallest difference pertains to the average neuroticism level between clusters.

### 3.2. Multivariate Analysis

The homogeneity of covariance was examined via the Box M test, and the null hypothesis of data fit was rejected (MBox = 1.63, F = 0.54, *p* > 0.05). Multivariate comparison (MANOVA) revealed significant differences between the two clusters (Wilks’ lambda = 0.89, F_(1,227)_ = 14.75, *p* < 0.001, η^2^ = 0.12). After the results of the multivariate test were analyzed, significant differences among clusters 1 and 2 were found for both dependent variables (WE and AE). The results are shown in Table 4. Cluster 1 had significantly higher levels of both WE and AE compared to Cluster 2.

### 3.3. Regression Analysis

To test the unique contribution of personality traits in predicting both WE and AE, a regression analysis was conducted. This analysis aimed to establish the unique relationship between each personality trait and the two outcomes while controlling age and gender. In both cases, age and gender were added in the first step, and in the second step five personality traits were added. The results of the regression analysis are shown in Table 5.

Age and gender were not significant predictors of WE. Adding the five personality traits significantly increased the explained variance in WE (F_(7,222)_ = 7.75, *p* < 0.001). Five personality traits explained 13% of the variance. Agreeableness and conscientiousness were significant, positive predictors of WE. Higher reported agreeableness (β = 0.26, *p* = 0.001) and conscientiousness (β = 0.14, *p* = 0.04) were related to higher levels of WE.

For AE, age and gender were both significant predictors in the first step (F_(2,222)_ = 6.99, *p* = 0.001) but lost significance after personality traits were added. The regression model with five personality traits and two control variables was statistically significant (F_(7,229)_ = 6.28, *p* < 0.001) and personality traits explained 11% of the variance. In this case, only conscientiousness was a significant predictor of AE (β = 0.17, *p* = 0.01). Participants with higher conscientiousness tend to have higher AE.

In summary, the regression analyses indicated that personality traits, particularly agreeableness and conscientiousness, were significant predictors of WE, while conscientiousness alone predicted AE. Overall, both models explained a low-to-modest amount of variance in the criteria. These findings suggest that certain personality characteristics may play a notable role in both outcomes.

## 4. Discussion

This study aimed to establish the relationship between personality traits and WE/AE in a sample of employed nursing students via a variable-centric (regression analysis) and a person-centric (cluster analysis) approach. It also aimed to examine the unique contribution of five personality dimensions on both WE and AE. Regarding the first research problem, the two emerged clusters are fully in line with similar, previous research [18,19]. The clusters have the same structure regarding the levels of the five personality traits, while the importance of the input for each trait differs. For example, Villafañe et al. [19] found that conscientiousness and neuroticism were the most relevant traits in their cluster analysis, while in this study, conscientiousness and extraversion were the most important personality traits regarding the order of the input. In order to answer the second research question, a multivariate analysis of variance revealed that the personality profile characterized by lower levels of neuroticism and higher levels of other four personality traits had higher WE and AE. The fact that the adaptive cluster had higher levels of both AE and WE supports the idea that our personality traits are reflected in many different situations and contexts [8]. If personality traits were not stable across contexts, we would expect different patterns in how they relate to WE and AE across studies. The clustering method used on personality dimensions is not without limitations. For instance, the silhouette coefficient in this study was 0.40, which indicates only a moderate level of separation between the two clusters, suggesting that the distinction between them is not particularly strong. Moreover, clustering results can be influenced by individual response biases. Some participants may evaluate themselves more negatively or positively due to factors like low self-esteem or social desirability, which can affect the reliability of the cluster formation and the interpretation of personality profiles.

Lastly, a regression analysis was performed in order to establish the unique contribution of the five personality dimensions in explaining WE and AE. It yielded similar results for WE and AE. In both models, adding personality traits leads to a small increase in the explained variance of the outcome. Higher conscientiousness was a significant predictor of both WE and AE, while higher agreeableness was related only to higher WE. Although some researchers reported significant relationships between other Big Five personality traits and WE/AE as well [38,39], they also reported a positive relationship between conscientiousness, agreeableness and these outcomes. Therefore, the results are generally in concordance with existing research. Individuals with higher conscientiousness usually exhibit a heightened sense of responsibility, organization, and diligence, which may influence their motivation to achieve both professional and academic goals.

Although a similar pattern of results for AE and WE is visible, a small amount of explained variance needs to be considered. Other factors, besides personality traits, also play a role in one’s AE and WE. An additional point worth noting is the distribution of agreeableness in our sample. The scores were positively skewed, with a substantial portion of participants rating themselves as highly agreeable. While such a distribution may raise concerns about response bias or social desirability effects, it is also plausible given the nature of the profession. Nursing, as a field, tends to attract individuals who are more empathetic, cooperative, and sensitive to the needs of others [38,40]. Additionally, some studies have found that females are generally more agreeable than males [41,42], and since this sample consists predominantly of female students, this may partly explain the skewed distribution. Therefore, the elevated levels of agreeableness observed in this sample may not necessarily reflect inflation, but rather an authentic representation of the personality profile common among nursing students and professionals.

There are several limitations to this study. First limitation is the overrepresentation of female students. Although 86.1% of the sample is female, the study sample is representative for nursing students at University North [43]. Across the world, the female gender is prevalent in nursing [44] and Croatia is not an exception to that rule. According to Statista [45], nursing professionals are 86% female in North America and 84% in Europe. The gender disparity is lowest in the African region (65% female), which is in line with the finding that gender stereotypes regarding professional choices tend to be more pronounced in more developed, wealthier countries [46]. As nursing remains a predominantly female profession globally, more research is needed to understand how these personality dynamics play out across gender lines and diverse cultural contexts. Future studies could focus on male nursing students as this study’s low representation of male students limits inferences about the personality dimensions and WE/AE of male professionals. The second limitation is the sample size—a larger number of participants may improve the validity and generalizability of the results. Some comparable studies, like Martos Martínez et al. [18], had a bigger sample size (N = 1268), while Villafañe et al. [19] had 90 participants. The third limitation relates to the self-report method used in this study. Social desirability in the academic setting, with a professor present, may have led students to overestimate traits typically valued in nursing, such as agreeableness and conscientiousness. The average agreeableness in the sample was M = 4.37 (SD = 0.66), and average conscientiousness was M = 4.10 (SD = 0.67). Personality traits are normally distributed in the general population [8], so the mean values for agreeableness and conscientiousness in our sample can be described as above average. However, one could argue that participants’ self-assessments were not influenced by social desirability and that they accurately assessed their personality traits as higher levels of agreeableness and conscientiousness are expected in a sample of nursing professionals. Moreover, the use of the same questionnaire (UWES-9 and UWES-9-S) to measure both dependent variables presents both a strength and a limitation. While it allows for comparison between the two types of engagement, it also increases the risk of common method variance. For example, participants might overestimate their WE, which could also inflate their AE scores. Consequently, the high correlation between WE and AE might reflect response biases rather than a true relationship between the two types of engagement. Lastly, this study employed a repeated cross-sectional descriptive predictive design so inferences about possible causality between the variables cannot be made.

Finally, a significant strength of this research is the inclusion of a unique sample of nursing professionals who are also university students. This dual-role perspective is rarely addressed in existing research, which typically focuses either on professionals or on students, but not both simultaneously. Additionally, examining both academic and work engagement within a single, dual-role sample represents a novel contribution to the literature. Understanding the nuanced relationships between personality traits and engagement in various contexts provides nursing institutions with insights for recruitment and training strategies. Our findings highlight the role of conscientiousness and agreeableness in predicting engagement. Although these traits are relatively stable [8], targeted interventions such as coaching for time management or team-based learning can help support behaviors aligned with these traits. By fostering environments that enhance these personality traits, institutions can improve nurse retention, reduce burnout, and maintain high standards of patient care. Interestingly, the observed correlation between AE and WE (r = 0.59, *p* < 0.01) suggests that engagement may be more trait-driven than environment-driven. However, the self-assessments of the two engagement types are quite congruent. Participants have the same academic environment, but they work in different institutions and environments. Given the variability in participants’ workplaces but uniformity in their academic setting, this moderate correlation underscores the potential role of individual dispositions over contextual influences. For example, two students with similar AE levels could have different WE assessments because one student might have a very stimulating work environment, whereas the other student might work in a non-stimulating environment. Considering the stated correlation, this is not the case; it seems that an individual’s engagement towards work or studying is largely based on the characteristics of an individual. Future studies might expand this line of inquiry by examining additional individual factors like mental health, grit, or cognitive abilities. These insights can inform strategies in nursing education and workforce development, ensuring support systems account for the diverse personalities within the profession.

## 5. Conclusions

This study aimed to analyze the relationships between the Big Five personality traits, WE and AE among employed nursing students. To identify personality profiles and determine whether there are significant differences among profiles in WE and AE, a cluster analysis was performed. The analysis yielded two clusters, named the adaptive and maladaptive cluster of personality (adaptive—a lower level of neuroticism and higher levels of the other four personality traits and maladaptive—the opposite). Participants in the adaptive cluster had higher levels of WE and AE. To analyze how the personality traits are uniquely related to WE and AE, a regression analysis was performed. Conscientiousness emerged as a significant, positive predictor of both AE and WE, while agreeableness was a significant, positive predictor of WE.

## Figures and Tables

**Table 1:** Descriptive statistics for all the study variables.

Personality Trait	Min	Max	Mean	SD	N	α
Extraversion (E)	1	5	363	0.79	230	0.65
Agreeableness (A)	1	5	4.37	0.66	230	0.66
Conscientiousness (C)	1.67	5	4.10	0.67	230	0.56
Neuroticism (N)	1	5	2.94	0.83	230	0.64
Openness (O)	1.33	5	3.77	0.67	230	0.60
WE	0.56	6	3.72	1.30	230	0.93
AE	0	6	3.11	1.46	230	0.96
WE—vigor	0	6	3.26	1.45	230	0.96
WE—dedication	1	6	4.09	1.34	230	0.88
WE—absorption	0	6	3.82	1.43	230	0.85
AE—vigor	0	6	2.56	1.56	230	0.93
AE—dedication	0	6	3.67	1.53	230	0.89
AE—absorption	0	6	3.09	1.57	230	0.90

Note: WE—work engagement, AE—academic engagement.

**Table 2:** Bivariate correlations among the main study variables.

	E	A	C	N	I	WE	AE	Age	Gender
E	1								
A	0.39 **	1							
C	0.24 **	0.37 **	1						
N	−0.29 **	−0.21 **	−0.18 **	1					
I	0.32 **	0.25 **	0.33 **	−0.09	1				
WE	0.16 *	0.33 **	0.26 **	−0.16 *	0.16 *	1			
AE	0.23 **	0.27 **	0.30 **	−0.14 *	0.26 **	0.59 **	1		
age	0.20 **	0.11	0.10	−0.17 **	18 **	0.05	18 **	1	
gender	0.08	0.37 **	19 **	0.02	0.08	0.09	0.16 *	0.02	1

Note: ** *p* < 0.01, * *p* < 0.05, E—extraversion, A—agreeableness, C—conscientiousness, N—neuroticism, O—openness, WE—work engagement, AE—academic engagement.

**Table 3:** Mean scores of personality traits for the total sample compared to those of the extracted clusters.

		Cluster	
	Total (N = 230)	1 (N = 136)	2 (N = 94)
	M (SD)	M (SD)	M (SD)
Conscientiousness (C)	4.10 (0.67)	4.43 (0.49)	3.61 (0.60)
Extraversion (E)	3.63 (0.79)	3.97 (0.68)	3.13 (0.65)
Agreeableness (A)	4.37 (0.66)	4.65 (0.42)	3.95 (0.73)
Openness (O)	3.77 (0.67)	4.04 (0.55)	3.37 (0.64)
Neuroticism (N)	2.94 (0.83)	2.50 (0.76)	3.31 (0.80)

Note: 1—adaptive cluster, 2—maladaptive cluster.

**Table 4:** Multivariate analysis of variance (effects between participants per cluster) depending on levels of WE and AE.

	Cluster 1		Cluster 2				
	M	SD	M	SD	F	*p*	η^2^
WE	4.02	1.22	3.28	1.30	19.66	<0.001	0.08
AE	3.49	1.40	2.55	1.37	25.64	<0.001	0.10

Note: η^2^—eta square, WE—work engagement, AE—academic engagement, 1—adaptive cluster, 2—maladaptive cluster.

**Table 5:** Five personality traits as predictors of WE and AE.

	WE		AE	
	Step 1	Step 2	Step 1	Step 2
	β	β	β	β
Age	0.04	−0.02	0.18 **	0.11
Gender	0.09	−0.04	0.16 *	0.08
Extraversion		−0.01		0.08
Agreeableness		0.26 **		0.10
Conscientiousness		0.14 **		0.17 *
Neuroticism		−0.08		−0.04
Openness		0.05		0.12
F_(df1,df2)_	1.08_(2,227)_	7.75_(7,222)_	6.99_(2,227)_	5.70_(7,222)_
R^2^	0.01	0.14	0.05	0.14
ΔR^2^		0.13		0.11

Note: ** *p* < 0.01, * *p* < 0.05, WE—work engagement, AE—academic engagement.

## Data Availability

The data presented in this study are available upon request from the corresponding author.

## Public Involvement Statement

There was no public involvement in any aspect of this research.

## Guidelines and Standards Statement

This manuscript was drafted against the JARS-Quant (Journal Article Reporting Standards) for Quantitative Research (American Psychological Association, 2020).

## Abbreviations

The following abbreviations are used in this manuscript:

WE	Work engagement
AE	Academic engagement
